# Evaluation of the changes in composition of pork chops during cooking

**DOI:** 10.1093/tas/txaa154

**Published:** 2020-08-18

**Authors:** Katelyn N Gaffield, Emily D Schunke, Jessica E Lowell, Anna C Dilger, Bailey N Harsh

**Affiliations:** Department of Animal Sciences, University of Illinois, Urbana-Champaign, IL

**Keywords:** cook loss, degree of doneness, intramuscular fat, marbling, tenderness

## Abstract

The objective was to determine the change in extractable lipid concentration during cooking of boneless pork chops to different endpoint temperatures. Pork loins (152 total) were used and three consecutive chops were cut from each loin. Chop 1 was evaluated raw (not cooked) for intramuscular fat (IMF) percentage. Raw IMF percentages were used to categorize the remaining two chops, from each loin, into low, average, and high marbling bins. The low bin included ≤3% IMF, the average bin included 3–4% IMF, and the high bin included ≥4% IMF. Chop 2 was cooked to 63 °C and chop 3 was cooked to 71 °C to evaluate cook loss, Warner–Bratzler shear force (WBSF), and IMF percentage. When cooked to either 63 or 71 °C, high IMF chops maintained greater (*P* < 0.001) IMF percentage than average and low IMF chops. Additionally, average IMF chops maintained greater (*P* < 0.001) IMF percentage than low chops, regardless of endpoint cooking temperature. The three marbling categories did not differ in cook loss (*P* = 0.28) or WBSF (*P* = 0.23) when chops were cooked to either 63 or 71 °C. However, both WBSF (2.76 kg) and cook loss (18.72%) were decreased (*P* < 0.001) in chops cooked to 63 °C compared with chops cooked to 71 °C (3.08 kg, 23.45%). Overall, differences in IMF percentages persisted even after cooking. Furthermore, IMF percentage of pork chops did not affect tenderness.

## INTRODUCTION

Consumers use both color and marbling as initial indicators of quality when visually evaluating pork products for purchase (Wood et al., 2004). Meat purchasing decisions are influenced by color more than any other attribute with consumers associating color with freshness or wholesomeness (Mancini and Hunt, 2005). Even so, consumers consider marbling to be most indicative of overall eating experience, including tenderness, juiciness, and flavor (Aberle et al., 2001; Rincker et al., 2008). However, several studies indicate that raw visual marbling is not necessarily predictive of sensory tenderness, juiciness, or flavor (Rincker et al., 2008; Wilson et al., 2016). A disconnect between the marbling consumers use to visually evaluate a pork chop in the store and actual intramuscular fat (IMF) percentage present in pork chops after cooking may explain this lack of association between IMF and tenderness.

Intramuscular fat concentration of pork chops changes during the cooking process. When cooked to 63 °C, water or moisture present in chops is lost, thereby concentrating extractable lipid or fat content (Unklesbay et al., 1983; Heymann et al., 1990). When chops are cooked to 71 °C, additional moisture is lost, and it is also possible that lipid is lost as well. With this potential loss of lipid, the difference in IMF between low and high marbled chops may narrow after cooking. A decrease in IMF after cooking may cause a decrease in perceived tenderness. If true, consumer predications of palatability from raw IMF appraisal may not result in the expected quality eating experience. It was hypothesized that IMF of cooked pork chops would be concentrated after cooking due to moisture loss and that possible lipid loss may narrow the range of variability in IMF compared to raw pork chops. It was additionally hypothesized that potential lipid loss would be greater in pork chops cooked to 71 °C than those cooked to 63 °C. Thus, the objective was to determine the change in extractable lipid concentration during cooking of boneless pork chops to different endpoint temperatures and assess the influences of IMF on tenderness.

## MATERIALS AND METHODS

Loins were sourced from a live animal trial that was reviewed and approved by the Institutional Animal Care and Use Committee at the University of Illinois.

### Carcass Fabrication

Loins (152 total) in the present study were collected from Duroc-sired pigs sourced from the University of Illinois Meat Science Laboratory (Urbana, IL). Production and slaughter practices were held constant for all originating pigs to reduce variability in loin quality traits associated with different production systems and slaughter conditions. Loins were fabricated following the specifications outlined in the North American Meat Processors Meat Buyer’s Guide (North American Meat Institute, 2014). Both anterior and posterior portions were fabricated to meet the specifications of a NAMP #414 Canadian back loin. After 1 d postmortem fabrication was complete, the anterior portions of the loins were vacuum packaged and aged at 4 °C for 13 d.

### Chop Collection

Three chops from each loin were removed posterior to the 10th rib cut surface. Chops were cut into 2.54-cm thick chops using a Bizerba deli slicer SE 12 D US (Bizerba USA Inc. Piscataway, NJ). Chop 1, from each loin, was trimmed of subcutaneous fat and secondary muscles, packaged in a Whirl-Pak bag (Nasco, Ft. Atkinson, WI), and stored at −2 °C until the evaluation of proximate composition. Both chops 2 and 3 were vacuum packaged and stored at −2 °C prior to cooking. Chop 1, closest to the 10th rib cut surface, was used for the determination of raw proximate analysis and subsequent categorization of chops 2 and 3 into appropriate marbling bins. Marbling bins were based on raw IMF percentage; low (≤3% IMF), average (3–4% IMF), or high (≥ 4% IMF). In total, the low bin contained 34 loins, the average bin contained 60 loins, and the high bin contained 58 loins. Furthermore, subjective marbling scores (National Pork Producers Council, 1999) were recorded on chop 1 for each loin on day 13 postmortem. Low IMF binned loins had an average subjective marbling score of 2.1, a range of 3.0 marbling score units, and an SD of 0.63 marbling score units. Average IMF binned loins had an average subjective marbling score of 2.6, a range of 2.5 marbling score units, and an SD of 0.60 marbling score units. High IMF binned loins had an average subjective marbling score of 3.2, a range of 2.0 marbling score units, and an SD of 0.46 marbling score units. Chop 2 was used to determine cook loss, Warner–Bratzler Shear Force (WBSF), and cooked proximate analysis (moisture and extractable lipid) when cooked to 63 °C. Chop 3 was used to determine these same traits when cooked to 71 °C.

### Raw Chop Proximate Analysis

Chop 1 was thawed at 25 °C and then homogenized using a Cuisinart (East Windsor, NJ) food processor. For each chop, duplicate homogenized 10-g samples were placed in a 110-°C drying oven for a minimum of 24 h. The chloroform–methanol solvent method outlined by Novakofski et al. (1989) was used to determine moisture and extractable lipid.

### Cook Loss and WBSF

Prior to analysis, chops 2 and 3 were thawed at 1 °C for approximately 24 h. Chops were removed from packaging and weighed before cooking. Chops were cooked on a Farberware Open Hearth grill (model 455N, Walter Kiddle, Bronx, NY) and internal temperature was monitored using copper-constantan thermocouples (Type T, Omega Engineering, Stamford, CT) connected to a scanning thermometer (model 92000-00, Barnat Co, Barrington, IL). The thermocouple was placed in the geometric center of each pork chop and monitored during cooking. Chop 2 was cooked, on one side, to an internal temperature of 31.5 °C, then flipped and cooked until a final internal temperature of 63 °C. Chop 3 was cooked, on one side, to an internal temperature of 35.5 °C, then flipped and cooked to a final internal temperature of 71 °C. After cooking, chops were allowed to cool to approximately 25 °C, and then weighed. Cook loss, of each pork chop, was calculated as: [(Initial weight, g c cooked weight, g)/initial weight, g] × 100. Five cores, approximately 1.25 cm in diameter, were removed from each chop parallel to the muscle fiber orientation. Cores were sheared using a Texture Analyzer TA.HD Plus (Texture Technologies Corp., Scarsdale, NY/Stable Mirosystems, Godalming, UK) with a load cell capacity of 100 kg and a blade speed of 3.33 mm/s. Shear force values of the five cores were averaged and reported as WBSF.

### Cooked Chop Proximate Composition

After coring, the remainder of the cooked chops (chops 2 and 3) were trimmed of all subcutaneous fat. The cooked chops were then individually homogenized, packaged in Whirl-Pak bags, and stored at −2 °C until the evaluation of cooked proximate composition using the same method as raw chop proximate composition.

### Statistical Analysis

Data were analyzed as a split-plot design in the MIXED procedure of SAS 9.4, (SAS Inst. Inc., Cary, NC) with the whole plot of marbling category and the split plot of degree of doneness. The model contained the fixed effects of marbling category and degree of doneness. The whole plot factor of marbling category was tested with the error term of (pig × marbling category). Mean, variance, and coefficient of variance (CV) of variables were calculated using the MEANS procedure. Homogeneity of variance was tested on raw data using the Levene’s test of the GLM procedure and variances were considered different at *P* ≤ 0.05. Box and whisker plots were used to demonstrate differences in variances. The bottom line of the box represents quartile 1 (Q1; 25th percentile); the middle line is the median (50th percentile); and the top line indicates quartile 3 (Q3; 75th percentile). Interquartile range (IQR) was calculated as Q3 − Q1. An upper fence was calculated as Q3 + (1.5 × IQR) and the lower fence was calculated as Q1 − (1.5 × IQR). Observations outside the upper and lower fences were considered outliers. The *P*-values represent differences in variances determined by the Levene’s test. Coefficient of variation is also provided representing variability that considers the magnitude of differences in the means.

## RESULTS AND DISCUSSION

Tenderness and juiciness are two of the main factors consumers use to evaluate eating experience (Moeller et al., 2010). Furthermore, when making pork-purchasing decisions, consumers base their assumptions of expected eating experience on visual marbling and often associate a greater amount of visual marbling with a more tender, juicy, and flavorful product (Wood et al., 2004; Lonergan et al., 2007). However, recent findings indicate that this may not be the case; visual marbling may not be as indicative of tenderness, juiciness, or flavor as consumers believe (Rincker et al., 2008; Wilson et al., 2016; Lowell et al., 2018). As a result, there seems to be a disconnect between methods consumers use to select pork products and factors affecting the quality of the final cooked product.

In the present study, raw percentage IMF was different (*P* < 0.001) between the low (2.47% IMF), average (3.52% IMF), and high (4.94% IMF) marbling bins, with a difference of 1.05 units IMF between the low and average bins and a difference of 1.42 units IMF between the average and high bins. Loins evaluated in the present study represented above average subjective marbling scores compared with industry-wide benchmarking data. Current literature surveying U.S. loin quality has reported average subjective marbling scores ranging from 2.27 to 2.43 (Wright et al., 2005; Moeller et al. 2010; Newman, 2017). Some published literature suggests potential differences in sensory tenderness and juiciness when IMF exceeds 3% (Brewer et al., 2001; Font-i-Furnols et al., 2012). When chops were cooked to 63 °C, cooked percentage IMF differed (*P* < 0.001) between the low (3.39% IMF), average (4.17% IMF), and high (5.59% IMF) marbling bins, with a difference of 0.78 units IMF between low and average bins and 1.42 units IMF between the average and high bins. When cooked to 71 °C, differences persisted (*P* < 0.001) between the low (3.88% IMF), average (4.55% IMF), and high (6.12% IMF) marbling bins with numerical differences of 0.67 units IMF between the low and average bins and 1.57 units between the average and high. In contrast to our hypothesis, this indicated that cooking chops to a higher endpoint temperature did not decrease differences in IMF between low, average, and high bins.

**Figure 1.**
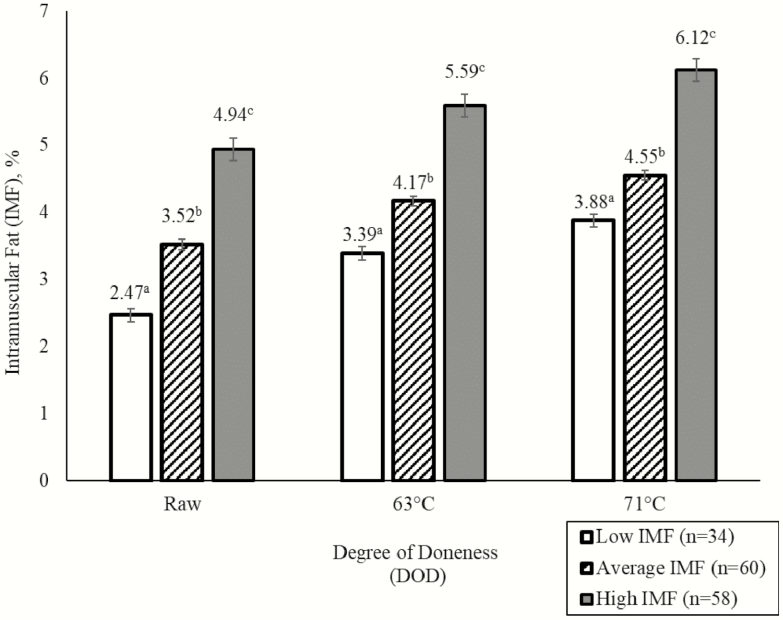
Effects of initial raw IMF bins and degree of doneness on final percentage IMF of pork loin chops. ^a,b,c^Bars within a degree of doneness category without a common superscript are different at (*P* ≤ 0.05).

During cooking, chop moisture is lost concentrating the proportion of extractable lipid. Similar to the findings of the current study, Heymann et al. (1990) demonstrated pork roasts cooked to 82 °C had 1.4% more extractable lipid compared to roasts cooked to 65 °C and also observed an increase in extractable lipid from 6.3% to 7.7% as cooking temperature increased from 65 to 82 °C. Additionally, moisture varied between the two endpoint temperatures as pork roasts cooked to 82 °C contained ~62% moisture, while roasts cooked to 65 °C contained ~66% moisture. Changes in extractable lipid were also observed in the present study as percentage extractable lipid increased (*P* < 0.001) with an increase in endpoint cooking temperature. Chops cooked to 71 °C had more (*P <* 0.001) extractable lipid than both the raw chops and chops cooked to 63 °C. Additionally, chops cooked to 63 °C had more (*P* < 0.001) extractable lipid than raw chops. Extractable lipid was concentrated (*P* < 0.001) by 0.75 units when raw chops were cooked to 63 °C and by 1.21 units when raw chops were cooked to 71 °C. Additionally, extractable lipid was concentrated (*P* < 0.001) by 0.46 units when endpoint temperature increased from 63 to 71 °C (Fig. 1). Although it was hypothesized that chops cooked to 71 °C would lose IMF in addition to moisture, this was not observed. No interaction was observed between initial marbling bin and degree of doneness (*P* = 0.10). Additionally, cooking chops to an endpoint temperature of 71 °C did not elicit differing levels of lipid loss between low, average, and high marbling bins to decrease variability in order to create a uniform eating experience.

**Figure 2.**
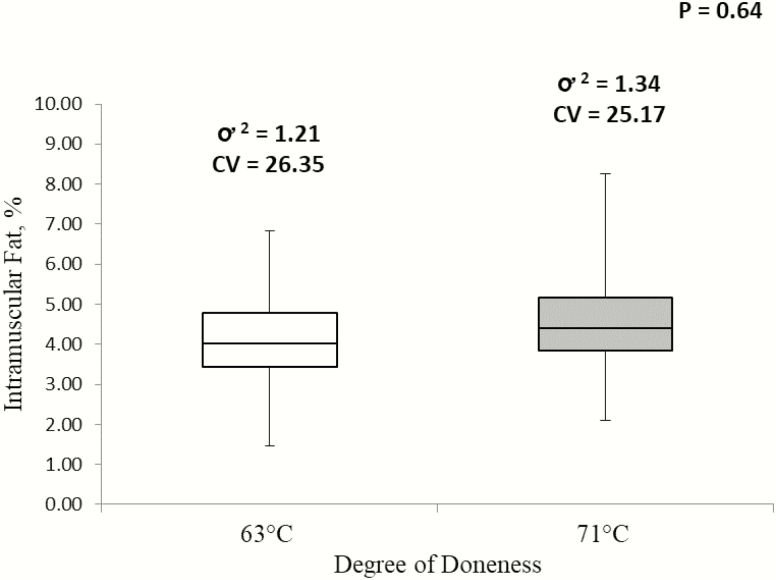
Variability of IMF percentage of chops cooked to 63 or 71 °C. CV represents calculated coefficient of variance and ơ ^2^ represents calculated variance.

**Figure 3.**
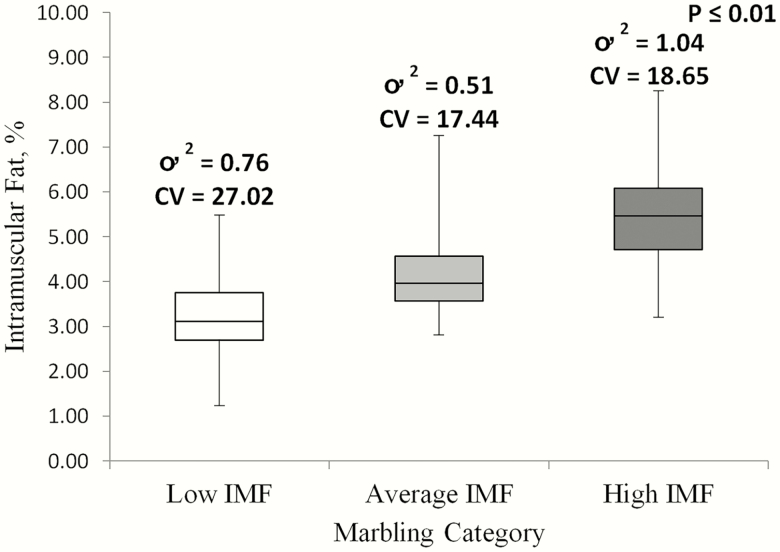
Variability of IMF percentage of cooked chops from low, average, and high raw IMF bins. CV represents calculated coefficient of variance and ơ ^2^ represents calculated variance.

Despite the initial hypothesis that chops cooked to 71 °C would be less variable in percentage IMF due to greater lipid loss, raw chops were less variable (*P* ≤ 0.001) in percentage IMF than pork chops cooked to either 63 or 71 °C (Fig. 2). Although variability in percentage IMF between chops cooked to either 63 and 71 °C did not differ (*P* = 0.64), there were differences (*P* ≤ 0.01) in variability observed between the low, average, and high bins (Fig. 3). High IMF chops demonstrated to be the most variable, while average IMF chops were the least variable.

**Figure 4.**
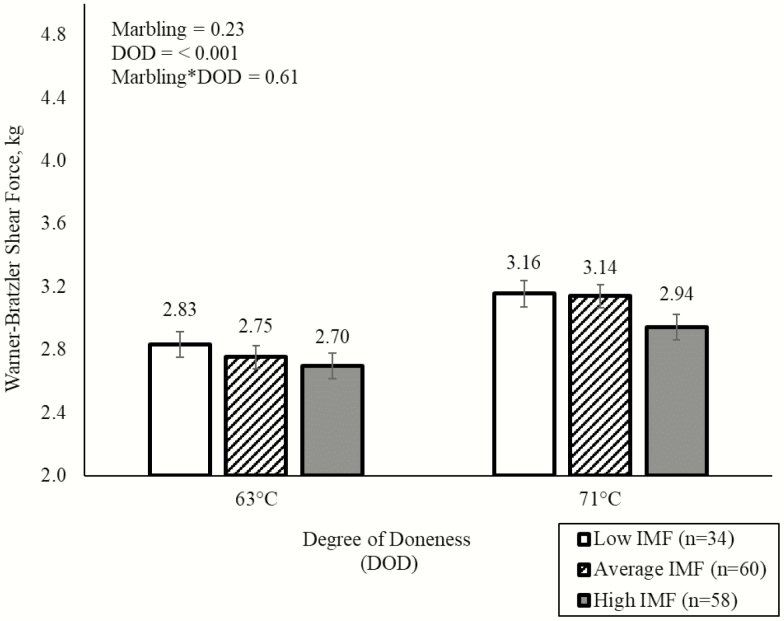
Effects of initial raw IMF bins and degree of doneness on Warner-Bratzler shear force values.

**Figure 5.**
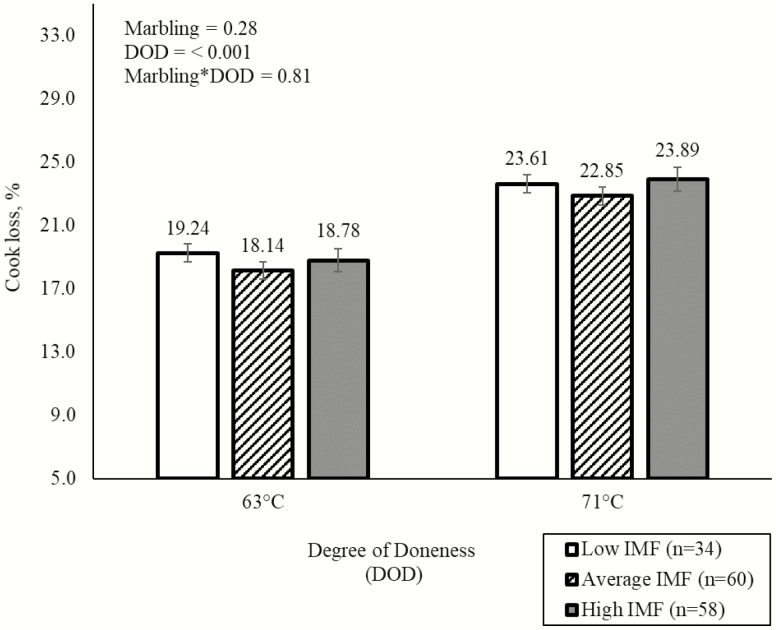
Effects of initial raw IMF bins and degree of doneness on cook loss.

Warner–Bratzler shear force was not different between marbling bins (*P* = 0.23; Fig. 4). No difference in WBSF was observed between high IMF chops and chops from the other two bins (*P* = 0.10). Similar to WBSF, there were no differences in cook loss (*P* = 0.28) between the high, average, and low marbling bins (Fig. 5). However, chops cooked to 63 °C were more tender (2.76 vs. 3.08 kg; *P* < 0.001) than chops cooked to 71 °C. Furthermore, chops cooked to an endpoint temperature of 71 °C experienced greater cook loss (a nearly 5-unit increase) than chops cooked to 63 °C (*P* < 0.001). Therefore, degree of doneness had a greater effect on tenderness than initial raw IMF percentage of pork chops. The difference between degrees of doneness was expected as decreasing endpoint cooking temperature from 71 to 63 °C improved sensory juiciness and tenderness (Rincker et al., 2008; Moeller et al., 2010). Furthermore, Honegger et al. (2019) also determined that the final internal temperature of pork chops had a greater impact on overall eating experience than marbling. Although there was a significant difference between chops cooked to 63 and 71 °C, previous studies have shown that consumers can only detect differences in tenderness greater than 0.5 kg (Miller et al., 1995). Ultimately, the differences between the degrees of doneness in the present study may not have an influence on consumer perception. Furthermore, when looking at the present study, both chops cooked to 63 and 71 °C had values well within the threshold associated with a tender eating experience (Miller et al., 1995).

These observations are supported by existing data that indicate that marbling may not affect overall eating quality as much as consumers believe; rather, overall eating quality is more dependent on endpoint cooking temperature (Rincker et al., 2008; Richardson et al., 2018; Honegger et al., 2019). It is also likely that the lack of differences observed in this study are because percentage IMF may not be the single point of variation in sensory tenderness and juiciness but instead one of several factors that play a role in pork quality sensory traits (Huff-Lonergan et al., 2002). To this effect, a study by Wilson et al. (2016) demonstrated that percentage IMF only explained about 2% of the variation in palatability of pork chops cooked to 63 °C. Similarly, Rincker et al. (2008) reported that percentage IMF did not influence sensory tenderness of pork chops cooked to 71 °C.

In conclusion, differences in cooked IMF percentages, between marbling bins, persisted within the three degree of doneness categories (raw, 63 °C, and 71 °C). Based on this, differences in the concentration of percentage IMF is not affected by moisture or potential lipid loss when evaluating chops ranging from approximately 2% to 6% IMF when cooked on an open hearth grill. Furthermore, WBSF and cook loss did not differ between marbling bins within the three degree of doneness categories. However, regardless of marbling bin, chops cooked to 63 °C were more tender and had less cook loss than chops cooked to 71 °C. Ultimately, degree of doneness had a more pronounced effect on tenderness and cook loss than percentage IMF or initial, raw marbling bin. Therefore, consumers should prioritize degree of doneness, as overall eating quality is much more dependent upon endpoint cooking temperature rather than raw visual quality traits, such as marbling.
